# Comment on Spedicati et al. Hidden in the Genome: The First Italian Family with North Carolina Macular Dystrophy Carrying a Novel *PRDM13* and *CCNC* Duplication. *Biomedicines* 2025, *13*, 1904

**DOI:** 10.3390/biomedicines14061226

**Published:** 2026-05-29

**Authors:** Kent W. Small

**Affiliations:** Macula and Retina Institute, 411 N. Central Ave Ste 115, Glendale, CA 91203, USA; kentsmall@hotmail.com

We read the article by Spedicati et al., “Hidden in the Genome: The First Italian Family with North Carolina Macular Dystrophy Carrying a Novel *PRDM13* and *CCNC* Duplication,” with great interest [1]. The authors describe a novel 98 kb duplication involving *PRDM13* and *CCNC* associated with North Carolina macular dystrophy (NCMD), identified as the first Italian family diagnosed with NCMD. Their work expands the NCMD spectrum of mutations and family ancestry.

We would like to draw attention to an earlier report by Manes et al., which describes a family with NCMD carrying a *PRDM13*-related duplication identified through whole-genome sequencing [2]. Although that pedigree was described in the article as a French family in Grenoble, France, the family’s origins were from Northern Italy. However, Manes et al. failed to mention the Italian heritage in their publication and there was no possible way for Spedicati et al. to be aware of this. We knew of this family’s Italian heritage because we had ascertained this exact same family in Grenoble, France, in 1996. The family history was personally and directly ascertained by KWS from the family members, which was also consistent with their Italian surname. Although Manes et al. ascertained the family after us, they identified the mutation before we did.

We emphasize that this observation does not diminish the novelty of the *PRDM13* and *CCNC* duplication reported by Spedicati et al. To avoid possible misinterpretation in future citations, the statement might be more precisely phrased as “the first NCMD family ascertained in Italy carrying a novel *PRDM13* and *CCNC* duplication” or similar language that distinguishes between ancestry and country of examination. In the literature, there are now two Italian families with NCMD.

Thank you for the opportunity to contribute this clarification to the NCMD literature.
